# Microtopography effects on pedogenesis in the mudstone-derived soils of the hilly mountainous regions

**DOI:** 10.1038/s41598-024-62540-y

**Published:** 2024-05-25

**Authors:** Banglin Luo, Jiangwen Li, Jiahong Tang, Chaofu Wei, Shouqin Zhong

**Affiliations:** 1 College of Resources and Environment/Key Laboratory of Eco-Environment in Three Gorges Region (Ministry of Education), Southwest University, No. 2 Tiansheng Road, Beibei District, Chongqing, 400715 People’s Republic of China; 2 District Agro-Tech Extension and Service Center of Shapingba, Chongqing, 400000 China; 3 Key Laboratory of Arable Land Conservation (Southwestern China), Ministry of Agriculture, Chongqing, 400715 China; 4 State Cultivation Base of Eco-Agriculture for Southwest Mountainous Land, Southwest University, Chongqing, 400715 China

**Keywords:** Pedogenic characteristics, Physicochemical property, Mudstone, Microtopography, Mineralogy, Toposequence, Element cycles, Geochemistry, Mineralogy, Environmental impact

## Abstract

Topography is a critical factor that determines the characteristics of regional soil formation. Small-scale topographic changes are referred to microtopographies. In hilly mountainous regions, the redistribution of water and soil materials caused by microtopography is the main factor affecting the spatial heterogeneity of soil and the utilization of land resources. In this study, the influence of microtopography on pedogenesis was investigated using soil samples formed from mudstones with lacustrine facies deposition in the middle of the Sichuan Basin. Soil profiles were sampled along the slopes at the summit, shoulder, backslope, footslope, and toeslope positions. The morphological, physicochemical, and geochemical attributes of profiles were analyzed. The results showed that from the summit to the toeslope, soil thickness increased significantly and profile configuration changed from A–C to A–B–C. The total contents of Ca and Na decreased at the summit, backslope, and footslope, while the total contents of Al, Fe and Mg showed an opposite trend. On the summit and shoulder of the hillslope, weathered materials were transported away by gravity and surface erosion, exposing new rocks. As a result, soil development in these areas was relatively weak. In flat areas such as the footslope and toeslope with sufficient water conditions, the addition of weathered components and the prolonged contact between water, soil, and sediment led to further chemical weathering, resulting in highly developed characteristics. Microtopography may influence physicochemical properties, chemical weathering, and redistribution of water and materials, causing variations in pedogenic characteristics at different slope positions.

## Introduction

Soil, as an independent natural body, sustains the lives and reproduction of various creatures on the land surface, while also developing and changing under the control of the soil-forming environment. Pedogenesis refers to the evolution from the profile scale to the regional scale, encompassing significant changes in the soil under physical, chemical, or biological conditions^1^. Among the five major soil forming factors, topography does not contribute new substances to the soil formation process. However, it strongly influences the physicochemical properties of soils by affecting the redistribution of water, soil materials, and nutrients^2–4^. The variations in water characteristics, combined with intense erosion, impact the redistribution of soil materials and elements during soil formation. This ultimately results in variations in the level of soil development across different parts of the terrain^5–9^. Therefore, soils on steep terrain tend to have rather shallow, poorly developed profiles compared to soils on nearby, more level sites^10^. Furthermore, the large topographic relief also results in orographic rain and temperature variations, which can affect the water and temperature conditions, as well as vegetation distribution, eventually impacting pedogenesis.

Small-scale topographic changes are defined as microtopographies. Microtopography refers to the concept that are relative to macrotopography, with amplitudes markedly smaller than the hillslope or basin scales^11^. Compared to microtopography, the greater variation of climate conditions and parent material due to large-scale topography often results in spatial heterogeneity of soil types. For instance, in the subtropical mountains of southeast China, the soils exhibit a vertical zonal distribution with yellow soil, yellow–red soil, and red soil from the summit to the footslope of the mountains^12^. However, in a small-scale toposequence, the differences between soils are primarily a result of topographic effects. This is because the soils in the sequence typically share the same parent material and have similar conditions such as climate, vegetation, and time^10,13^. A series of toposequences derived from granite, sandstone-gneiss, limestone, and slate have shown that topography plays an essential role in pedogenesis^14–18^. Therefore, the difference between large- and micro-topography in the impact on soil formation process makes it necessary to explore the pedogenesis under the influence of small-scale terrain, especially in hilly mountainous regions with little relief. Compared to the equivalent background state, the presence of microtopography increases the proportion of rainfall infiltration^11^. Within the influence of microtopography, the rainfall distribution pattern and surface operation mode are completely different. In addition, microtopography can control a series of geomorphic processes, such as collapse, transportation, and accumulation, to alter the spatial redistribution of light, heat, water, nutrients, and soils in a small area, thus affecting vegetation growth^3,4,19^. The redistribution of water and soil material caused by microtopography is the main factor in soil spatial variation and an poses an obstacle to the utilisation of soil resources^13^, especially in hilly mountainous regions. Several studies have demonstrated the close relationship between small-scale landscape topography and soil physicochemical properties such as soil compactness index (bulk density, clay and clay/organic matter ratio)^7^, pH^3^, weathering degree^20–22^, clay mineral composition^23^, and microbial community abundance^4,24^. These factors should be considered in small-scale environmental analysis^13^. Specifically, microtopography characteristics are key factors that control soil pH in flysch regions across Switzerland by affecting pedogenesis processes and vegetation^3^. Pal et al.^25^ discovered that presence of microtopography led to the formation of non-alkaline and highly alkaline soils on the upper and lower slopes, respectively, of the southwestern Indo-Gangetic Plain. Botschek et al.^26^ noted that the organic matter content was highest in the mineral topsoil on the uphill slope, gradually decreasing towards the foot of the slope. The current research on the influence of topography on soil formation or soil type primarily focuses on large-scale topography^12^. The topography of hilly areas (with a relative height of less than 200 or 100 m) does not usually affect the higher classification types of soil. However, the redistribution of water and heat by microtopography can impact the lower classification types of soil, such as soil species. Soil species are directly related to soil fertility and land use. There are large hilly areas in the world, and the redistribution of water and temperature conditions by microtopography will have an important impact on soil formation. However, there are few relevant studies at present, so the study of microtopography on soil formation has broad significance.

The purple mudstone and sandstone in the middle of the Sichuan Basin were eroded by water, leading to the creation of hilly topography. Heat and water are redistributed in different locations of topography, combined with severe soil erosion, resulting in differences in particle size distribution, nutrient content, water holding capacity, drought resistance, and fertiliser retention. Therefore, the degree of soil development varies. Finding an effective solution for soil erosion and its related issues in this region is a significant challenge we are currently facing. Moreover, the mudstone-derived soils that develop from purple mudstone undergo rapid physical weathering but slow chemical weathering, resulting in the formation of soil particles (i.e., formation of a regolith) in approximately five years^27^. We hypothesize that in mudstone soil-forming regions with similar parent materials, comparable climatic conditions, minimal relief of topography, and rapid physical weathering rates, the impact of microtopography might be dominant. Despite numerous researches on soil water, nutrients, texture and erosion in Sichuan Basin^27^, there is a lack of research on soil formation characteristics of mudstone under the influence of microtopography. Therefore, we selected five toposequences of soil derived from mudstone in the hilly regions of Sichuan Basin to explore their physicochemical, morphological, and geochemical properties. The aim of the present study was to illuminate the weathering development and pedogenic characteristics of mudstone under the influence of microtopographic conditions, ultimately providing a basis for the management of mudstone soil in mountainous and hilly regions.

## Materials and methods

### Study area

**Figure 1:**
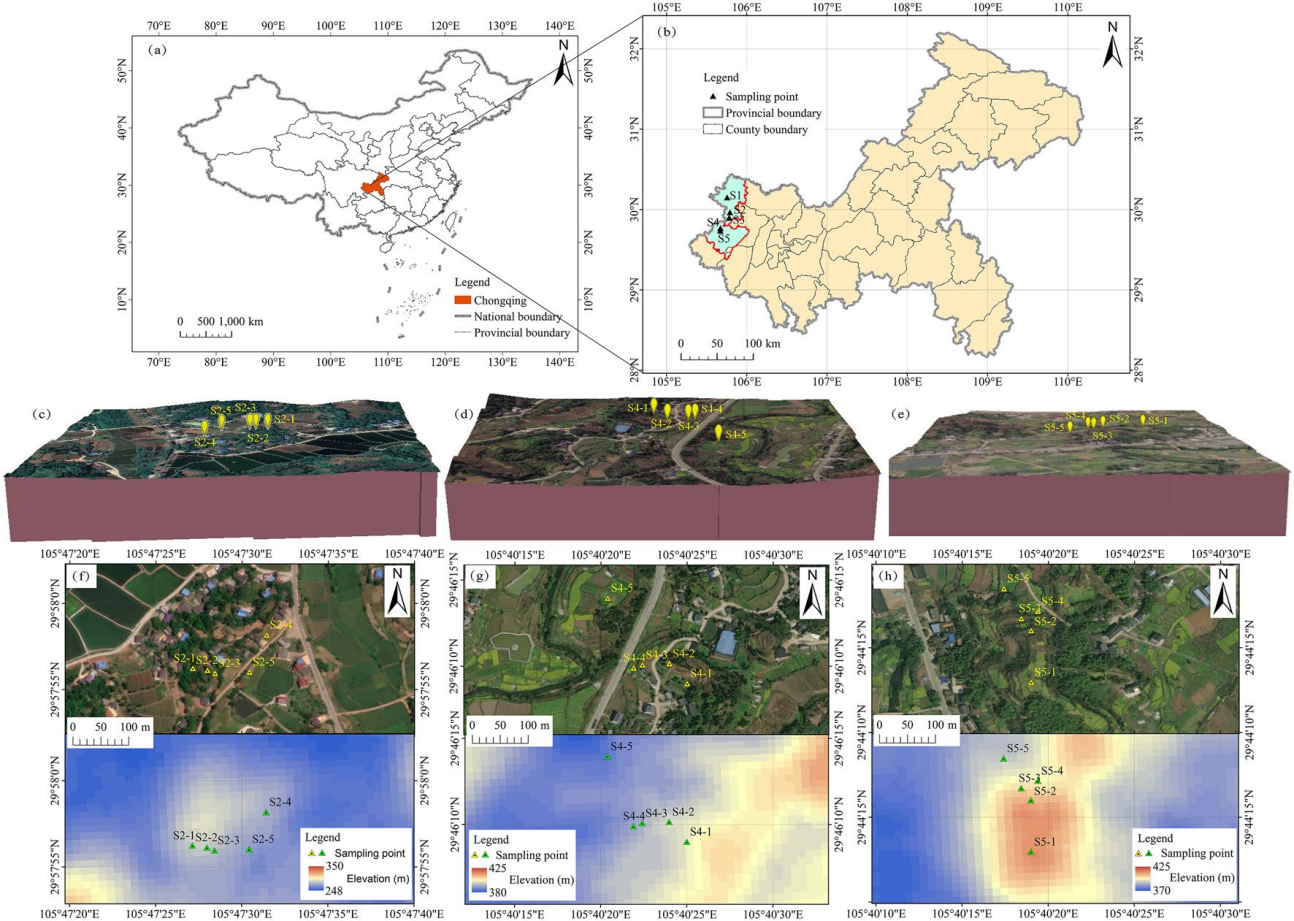
Study area (**a**, **b**), 3D models (**c**, **d**, **e**), and the satellite images and 12.5 m DEM (**f**, **g**, **h**) of sampling points. Take the S2, S4, and S5 as examples. The background DEM dataset was downloaded from Earthdata, NASA (https://search.asf.alaska.edu). The satellite images were obtained from Tianditu (https://map.tianditu.gov.cn).

The study area, Tongnan District and Dazu District, located in the central Sichuan Basin (Fig. 1). The area is characterized by predominantly hilly topography and a subtropical monsoon climate. Vegetation types in the study area are consist primarily of subtropical evergreen broad-leaved forests. The slope of the study area, calculated using a 12.5 m DEM, ranges from 0° to 69°, with a corresponding slope length factor varying from 0 to 55.64. In the period of 2001 to 2020, the multi-year average precipitation and temperature were 1125.4 mm and 18.1 °C, respectively. According to the third national land resource survey, the predominant land use types in the study area were cultivated land and forest land, both of which account for more than 65% of the total land area (http://ghzrzyj.cq.gov.cn, http://www.cqtn.gov.cn). The soils were classified as Cambisols in the WRB classification^28^.

### Soil sampling

**Figure 2:**
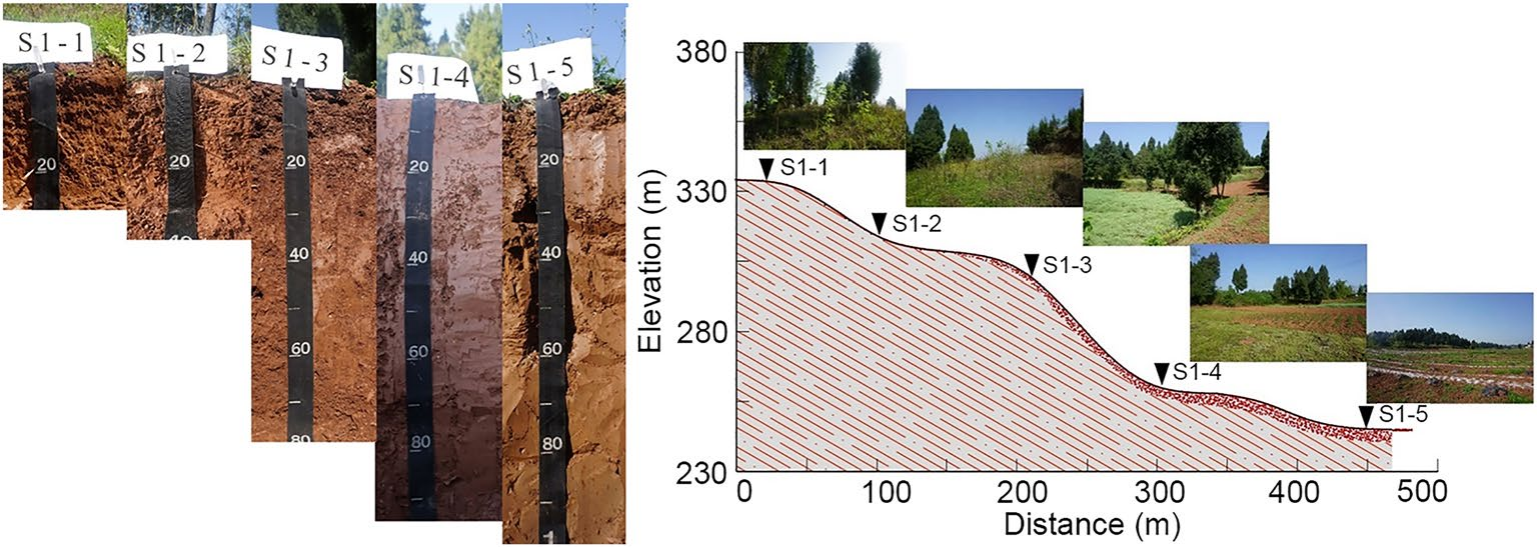
Soil profiles along a toposequence (S1-1, S1-2, S1-3, S1-4, and S1-5 represent the summit, shoulder, backslope, footslope, and toeslope positions of the hillslope, respectively. Take S1 as an example).

Five natural and typical toposequences were identified, and along each toposequence, five profiles were selected for sampling based on the microtopography conditions. The sampling points were detailed in Table 1. According to the 1:200,000 regional geological map of China (https://geocloud.cgs.gov.cn) and the field survey, the parent material of all the sampling points was purple mudstone of the Upper Jurassic Shaximiao Formation in Mesozoic. Additionally, in order to ensure uniformity, other soil-forming factors such as climate (specifically rainfall and temperature) and organisms (mainly grasses and crops as the predominant surface vegetation, with soil animals detailed in Table 2) were also investigated for each sampling point, in addition to the terrain (Table 1). Additionally, according to the formula reported by Caniani et al.^29^, the topographic index (TPI) of each sampling point was calculated by using a 12.5 m DEM, which reflected the flow accumulation of each sampling point (Table 1). A higher TPI indicates a larger accumulation of discharge, relatively low-lying terrain, and higher soil water content. Five soil samples with five repetitions each were collected at the summit, shoulder, backslope, footslope, and toeslope positions of the hillslope (Fig. 2). Soil samples were collected from the excavated soil profiles, sampling from the toeslope to the summit based on soil genesis characteristics, which were divided into horizons A, B, and C. The GPS locator was used to record the coordinates, altitude, and slope of the sampling points. Additionally, the morphological characteristics of the soil profiles were recorded according to the *Field Book for Describing and Sampling Soils (version 3.0)*^30^ (Table 2). Soil samples were taken to the laboratory for chemical, physical and mineralogical analyses, totalling 125 samples (approximately 500 g each). All the samples were air-dried. and then sieved through a 2 mm sieve after the removal of visible plant debris for laboratory analysis.

**Table 1:** The information of sampling points.

Sampling Point	Coordinates (N/E)		Elevation (m)	Slope gradient (°)	Average annual rainfall (mm)	Average annual temperature (°C )	TPI	Land use	Slope position
S1-1	30° 09′ 04″	105° 45′ 31″	331	10	1121.5	18.2	7.00	Grassland	Summit
S1-2	30° 09′ 04″	105° 45′ 32″	320	15	1121.5	18.2	9.93	Grassland	Shoulder
S1-3	30° 09′ 03″	105° 45′ 18″	307	10	1121.5	18.2	2.65	Dry land	Backslope
S1-4	30° 09′ 06″	105° 45′ 19″	265	0	1121.5	18.2	10.21	Paddy field to dry land	Footslope
S1-5	30° 09′ 04″	105° 45′ 16″	242	0	1121.5	18.2	7.92	Paddy field to dry land	Toeslope
S2-1	29° 57′ 56″	105° 47′ 27″	330	2	1126.9	18.3	4.70	Grassland	Summit
S2-2	29° 57′ 56″	105° 47′ 28″	328	2	1126.9	18.3	7.46	Grassland	Shoulder
S2-3	29° 57′ 56″	105° 47′ 28″	314	10	1126.9	18.3	2.17	Dry land	Backslope
S2-4	29° 57′ 58″	105° 47′ 31″	297	10	1126.9	18.3	7.46	Dry land	Footslope
S2-5	29° 57′ 56″	105° 47′ 30″	265	0	1126.9	18.3	2.18	Paddy field to dry land	Toeslope
S3-1	29° 53′ 46″	105° 46′ 59″	319	5	1130.3	18.5	6.98	Paddy field to dry land	Summit
S3-2	29° 53′ 46″	105° 46′ 59″	301	10	1130.3	18.5	9.46	Dry land	Shoulder
S3-3	29° 53′ 48″	105° 47′ 00″	298	6	1130.3	18.5	4.60	Dry land	Backslope
S3-4	29° 53′ 53″	105° 47′ 00″	267	0	1130.3	18.5	6.98	Dry land	Footslope
S3-5	29° 53′ 55″	105° 47′ 03″	240	0	1130.3	18.5	4.01	Paddy field to dry land	Toeslope
S4-1	29° 46′ 09″	105° 40′ 25″	408	2	1126.7	18.0	7.16	Grassland	Summit
S4-2	29° 46′ 10″	105° 40′ 24″	403	15	1126.7	18.0	10.68	Dry land	Shoulder
S4-3	29° 46′ 10″	105° 40′ 22″	392	20	1126.7	18.0	9.99	Dry land	Backslope
S4-4	29° 46′ 10″	105° 40′ 22″	396	0	1126.7	18.0	4.26	Dry land	Footslope
S4-5	29° 46′ 14″	105° 40′ 20″	385	0	1126.7	17.9	9.80	Dry land	Toeslope
S5-1	29° 44′ 13″	105° 40′ 19″	421	6	1127.6	18.0	8.50	Grassland	Summit
S5-2	29° 44′ 16″	105° 40′ 19″	406	15	1127.6	18.0	1.57	Dry land	Shoulder
S5-3	29° 44′ 17″	105° 40′ 18″	394	10	1127.6	18.0	3.87	Dry land	Backslope
S5-4	29° 44′ 17″	105° 40′ 19″	396	0	1127.6	18.0	4.73	Paddy field to dry land	Footslope
S5-5	29° 44′ 18″	105° 40′ 17″	387	0	1127.6	18.0	6.76	Paddy field to dry land	Toeslope

The average annual rainfall of the sampling points was calculated by the annual precipitation data of 1 km resolution in China (2001–2020) (National Earth System Science Data Center, National Science & Technology Infrastructure of China (http://www.geodata.cn)); The average annual temperature was calculated by the monthly mean air temperature raster data of China from 2001 to 2020 (1 km resolution)^31^.

**Table 2:** Morphological attributes of the soil profiles.

Profile No.	Horizon	Depth (cm)	Soil colour		Soil structure	Plasticity	Animal activity	Intrusions
			Dry state	Wet state				
S1-1	Ah	0–12	2.5YR 5/6	2.5YR 4/6	BS	Not plastic	Earthworm	–
	C	> 12	2.5YR 5/6	2.5YR 4/6	BS	Not plastic	–	–
S1-2	Ap	0–25	2.5YR 5/4	2.5YR 4/4	GS	Not plastic	–	–
	C	> 25	2.5YR 5/6	2.5YR 4/6	BS	Not plastic	–	–
S1-3	Ap	0–18	2.5YR 6/4	2.5YR 4/4	GS, BS	Not plastic	Ant nest	–
	Bw1	18–31/47	2.5YR 5/4	2.5YR 4/4	BS	Slightly plastic	–	Bricks and rubbles
	Bw2	31/47–72	2.5YR 5/4	2.5YR 4/4	BS	Slightly plastic	–	–
	C	> 72	2.5YR 5/6	2.5YR 4/6	BS	Not plastic	–	–
S1-4	Ap	0–12	2.5YR 5/3	2.5YR 4/3	BS	Slightly plastic	–	Cinders
	Bw1	12–45	2.5YR 5/3	2.5YR 4/3	BS	Medium plastic	–	Shells
	Bw2	45–70	2.5YR 5/3	2.5YR 4/3	BS	Medium plastic	–	–
	Bw3	70–90	2.5YR 5/3	2.5YR 4/3	BS	Medium plastic	–	–
	C	> 90	2.5YR 5/6	2.5YR 4/6	BS	Not plastic	–	–
S1-5	Ap	0–14	2.5YR 6/8	2.5YR 6/8	BS	Slightly plastic	–	Bricks and rubbles
	Bw1	14–30	2.5YR 6/8	2.5YR 5/8	BS	Medium plastic	–	Shells
	Bw2	30–58	2.5YR 6/8	2.5YR 5/8	BS	Medium plastic	–	–
	Bw3	65–100	2.5YR 6/8	2.5YR 5/8	BS	Plastic	–	–
S2-1	Ah	0–10	2.5YR 5/6	2.5YR 4/6	BS	Not plastic	–	–
	C	> 10	2.5YR 5/6	2.5YR 4/6	BS	Not plastic	–	–
S2-2	Ap	0–15	2.5YR 5/4	2.5YR 5/6	BS, GS	Not plastic	Earthworm, Centipede	–
	Bw	15–40	2.5YR 5/4	2.5YR 4/4	BS	Not plastic	–	–
	C	> 40	2.5YR 5/6	2.5YR 4/6	BS	Not plastic	–	–
S2-3	Ap	0–24	2.5YR 6/6	2.5YR 6/6	GS	Not plastic	Ant	–
	Bw1	24–35	2.5YR 6/6	2.5YR 5/6	BS, GS	Not plastic	Beetle	–
	Bw2	35–51	2.5YR 6/6	2.5YR 5/6	BS	Slightly plastic	–	–
	C	> 51	2.5YR 5/6	2.5YR 4/6	BS	Not plastic	–	–
S2-4	Ap	0–15	2.5YR 5/3	2.5YR 4/3	BS	Not plastic	–	Cinders
	Bw1	15–35	2.5YR 5/3	2.5YR 4/3	BS	Slightly plastic	–	Shells
	Bw2	35–62	2.5YR 5/3	2.5YR 4/3	BS	Medium plastic	–	–
	Bw3	62–95	2.5YR 5/3	2.5YR 4/3	BS	Medium plastic	–	–
	C	> 95	2.5YR 5/6	2.5YR 4/6	BS	Not plastic	–	–
S2-5	Ap	0–25	5YR 5/6	5YR 4/6	BS, GS	Slightly plastic	–	–
	Bw1	25–42	5YR 5/4	5YR 4/4	BS	Slightly plastic	–	Shells
	Bw2	42–70/74	5YR 5/4	5YR 4/4	BS	Medium plastic	–	–
	Bw3	70/74–100	5YR 5/4	5YR 5/4	BS	Medium plastic	–	–
S3-1	Ah	0–20/25	2.5YR 5/4	2.5YR 4/4	BS	Not plastic	–	–
	C	> 20/25	2.5YR 5/6	2.5YR 4/6	BS	Not plastic	–	–
S3-2	Ap	0–20	2.5YR 5/4	2.5YR 6/6	GS	Not plastic	Earthworm	–
	Bw1	20–33	2.5YR 5/4	2.5YR 6/6	GS	Not plastic	–	–
	Bw2	33–60	2.5YR 5/4	2.5YR 6/6	BS	Slightly plastic	–	–
	C	> 60	2.5YR 5/6	2.5YR 4/6	GS	Not plastic	–	–
S3-3	Ap	0–22	2.5YR 6/4	2.5YR 4/4	GS	Not plastic	–	–
	Bw1	22–37	2.5YR 5/4	2.5YR 4/4	BS, GS	Not plastic	–	Bricks
	Bw2	37–75	2.5YR 5/4	2.5YR 4/4	BS	Slightly plastic	–	–
	C	> 75	2.5YR 5/6	2.5YR 4/6	BS	Not plastic	–	–
S3-4	Ap	0–15	2.5YR 5/3	2.5YR 4/3	BS	Slightly plastic	Ant	–
	Bw1	15–38	2.5YR 5/3	2.5YR 4/3	BS	Slightly plastic	–	–
	Bw2	38–75	2.5YR 5/3	2.5YR 4/3	BS	medium	–	–
	Bw3	75–100	2.5YR 5/3	2.5YR 4/3	BS	Medium plastic	–	–
S3-5	Ap	0–18	5YR 5/6	5YR 4/6	BS	Slightly plastic	Earthworm	–
	Bw1	18–33	5YR 5/4	5YR 4/4	BS	Medium plastic	–	–
	Bw2	33–56	5YR 5/4	5YR 4/4	BS	Plastic	–	–
	Bw3	56–84	5YR 5/4	5YR 5/4	BS	Plastic	–	–
	Bw4	84–100	5YR 4/4	5YR 3/4	BS	Plastic	–	–
S4-1	Ah	0–20	10R 5/4	10R 4/4	BS	Not plastic	–	–
	C	> 20	10R 4/4	10R 3/4	BS	Not plastic	–	–
S4-2	Ap	0–25	10R 5/4	10R 4/4	GS	Not plastic	Earthworm	–
	Bw	25–40	10R 4/4	10R 3/4	GS	Slightly plastic	–	–
	C	> 40	10R 4/4	10R 3/4	BS	Not plastic	–	–
S4-3	Ap	0–20	2.5YR 5/6	2.5YR 4/6	BS	Not plastic	–	–
	Bw	20–40/55	2.5YR 5/6	2.5YR 4/6	BS	Medium plastic	–	–
	C	> 40/55	2.5YR 4/6	2.5YR 3/6	BS	Not plastic	–	–
S4-4	Ap	0–20	2.5YR 5/3	2.5YR 4/3	GS, BS	Not plastic	Ant	–
	AB	20–35	2.5YR 4/3	2.5YR 4/3	BS	Not plastic	–	–
	Bw1	35–51	2.5YR 4/3	2.5YR 4/3	BS	Medium plastic	–	Cinders
	Bw2	51–83	2.5YR 4/3	2.5YR 4/3	BS	Medium plastic	–	–
	C	> 83	2.5YR 4/4	2.5YR 3/4	BS	Not plastic	–	–
S4-5	Ap	0–20	2.5YR 5/3	2.5YR 4/3	BS, GS	Not plastic	Earthworm	–
	Bw1	20–45	2.5YR 5/3	2.5YR 4/3	BS	Medium plastic	–	Shells
	Bw2	45–68/75	2.5YR 5/3	2.5YR 4/3	BS	Medium plastic	–	–
	Bw3	68/75–100	2.5YR 4/3	2.5YR 4/3	BS	Medium plastic	–	–
S5-1	Ah	0–18	10R 5/6	10R 4/6	GS	Not plastic	–	–
	C	> 18	10R 4/6	10R 3/6	BS	Not plastic	–	–
S5-2	Ap	0–10/15	10R 5/6	10R 4/6	GS	Not plastic	Earthworm, Centipede	–
	AC	10/15–25	10R 5/6	10R 4/6	BS	Not plastic	–	–
	C	> 25	10R 4/6	10R 3/6	BS	Not plastic	–	–
S5-3	Ap	0–22	2.5YR 5/6	2.5YR 4/6	BS	Not plastic	–	–
	AB	22–45	2.5YR 5/6	2.5YR 4/6	BS	Slightly plastic	–	–
	Bw	45–65	2.5YR 5/6	2.5YR 4/6	BS	Medium plastic	–	Cinders
	C	> 65	2.5YR 4/6	2.5YR 3/6	BS	Not plastic	–	–
S5-4	Ap	0–10	2.5YR 5/3	2.5YR 4/3	BS	Slightly plastic	Ant nest	Cinders
	Bw1	10–30	5YR 6/4	5YR 4/6	BS	Medium plastic	–	Shells
	Bw2	30–55	2.5YR 4/3	2.5YR 4/3	BS	Medium plastic	–	–
	Bw3	55–68	2.5YR 4/3	2.5YR 4/3	BS	Plastic	–	–
	Bw4	68–100	2.5YR 4/4	2.5YR 3/4	BS	Plastic	–	–
S5-5	Ap	0–30	5YR 5/6	5YR 4/6	BS	Not plastic	Earthworm	–
	Bw1	30–55	5YR 5/4	5YR 4/4	BS	Medium plastic	–	Shells
	Bw2	55–77	5YR 5/4	5YR 4/4	BS	Medium plastic	–	–
	Bw3	77–100	5YR 5/4	5YR 5/4	BS	Plastic	–	–

BS, blocky structure; GS, granular structure.

### Experimental method and data analysis

#### Physical and mineralogical methods

The soil bulk density was determined using the soil core (volume = 100 cm^3^) method. Prior to conducting the soil particle size analysis (PSA), hydrogen peroxide was used to remove organic matter. The removal of iron oxide was achieved using a sodium bicarbonate-buffered, sodium dithionite-citrate system, while sodium acetate was utilized to remove carbonate. Sodium hexametaphosphate was used to disperse soil particles and PSA was determined by pipette method^32^.

Soil porosity (*φ*) can be calculated by the following formula^32^,

1 $$\varphi = \left( {1 - \frac{{\rho_{b} }}{{\rho_{p} }}} \right) \times 100$$

where *ρ*_*b*_ and *ρ*_*p*_ represent bulk density (g cm^−3^) and particle density, respectively. Particle density was determined by pycnometer method.

X-ray diffraction (XRD: D8 Advance X-ray diffractometer, Bruker, Germany) was utilized to analyze the mineral composition of soil samples. The soil samples were crushed to particle size < 1 mm, soaked in distilled water, and dispersed by ultrasonic wave. Carbonate and organic matter were removed from the samples, and the clay particles were absorbed by suspension centrifugation to settle. After centrifugation, the samples were dried at a temperature below 60 °C. The dried samples were ground with an agate mortar until the hands had no granular feeling, and then wrapped in paper for XRD determination. The X-ray diffractometer parameters were set as follows: the radiation source was CuKα, the scanning speed was set to 2° min^−1^, the sampling step width was 0.02°, and the scanning range was 5°–45°. The diffraction data was interpreted based on the X-ray diffraction pattern of soil samples, and then compared with known standard minerals data to identify the minerals types present in the soil. The percentage of minerals in the soil was calculated by using the integral intensity of diffraction peaks on the diffraction pattern and the reference intensity of common minerals^33^.

#### Chemical methods

A glass electrode was used to measure the soil pH in a 1:2.5 soil/water suspension ratio. Soil organic carbon (SOC) content was determined using the dichromate-wet combustion method, and the C/N ratio was calculated as the ratio of SOC to total nitrogen (TN) content (determined by the Kjeldahl method). Cation exchange capacity (CEC) was determined using the Na saturation method. Each sample (0.5 g) was ground to 100 mesh (0.15 mm) in an agate mortar and formed into a tablet to measure the geochemical elements of the test soil using X-ray fluorescence spectrometry^32,34^. These geochemical elements include macroelements, trace elements, rare-earth elements, and radioactive elements. The content and composition characteristics of macroelements are widely used as indicators. Therefore, the geochemical elements in this study refer to the 10 macroelements^35^.

The chemical index of alteration (CIA) is the measure of the degree of the feldspar-to-clay conversion, which is proportional to the clay mineral/feldspar ratio. During chemical weathering, alkali metals are leached out from feldspar in the form of ions, leading to the formation of clay minerals and simultaneous fluctuation in the molar content of Al_2_O_3_. Thus, CIA is defined as follows^36^,

2 $${\text{CIA}} = \left( {\frac{{{\text{Al}}_{2} {\text{O}}_{3} }}{{{\text{Al}}_{2} {\text{O}}_{3} + {\text{CaO}}^{*} + {\text{Na}}_{2} {\text{O}} + {\text{K}}_{2} {\text{O}}}}} \right) \times 100$$

where all variables represent the molar faction of macroelement oxides and CaO* is the molar faction of CaO associated with silicate minerals calculated using the McLennan method^37^. Generally, CIA values are below 50 for unweathering with feldspar, 50–65 for weak weathering with plagioclase, 65–85 for moderate weathering with illite and montmorillonite, and near 100 for strong weathering with kaolinite and chlorite. Additionally, CIA indirectly indicates the change in climate, with higher CIA values reflecting warmer and wetter climates.

The chemical index of weathering (CIW) is conceptually and computationally similar to CIA, except for the lack of K_2_O. Many scholars have found that the content of K in a sedimentary area is higher than that in provenance rock area, probably due to K metasomatism or the addition of eolian dust undergoing diagenetic processes. To eliminate the interference of diagenetic K, the concept of the CIW was proposed by Holail and Moghazi^38^,

3 $${\text{CIW}} = \left( {\frac{{{\text{Al}}_{2} {\text{O}}_{3} }}{{{\text{Al}}_{2} {\text{O}}_{3} + {\text{CaO}}^{*} + {\text{Na}}_{2} {\text{O}}}}} \right) \times 100$$

where the oxides are given in molar faction and CaO* is calculated by the same method of the CIA. Chemical weathering increase is indicated by high CIW values.

The Na/K molar ratio indicates the extent of chemical weathering of plagioclase. Plagioclase is rich in Na, especially compared to potassium feldspar, which is rich in K. Plagioclase undergoes a higher rate weathering compared to potassium feldspar^10,35^, resulting in an inverse relationship between chemical weathering intensity and the Na/K molar ratio^39^.

The migration coefficient of element X (*MC*_*X*_) represents the migration and enrichment characteristics of soil profile relative to bedrock during pedogenesis^40–42^. Titanium (Ti) is commonly used as a stationary component to determine the movement of other elements^40^, enabling for the inference of soil development degree and rate. The *MC*_*X*_ can be calculated by the following formula,

4 $$MC_{X} = \left( {\frac{{X_{soil} }}{{X_{rock} }} \times \frac{{Ti_{rock} }}{{Ti_{soil} }} - 1} \right) \times 100$$

where soil and rock subscripts in the formula represent the concentrations of elements X and Ti in soil and bedrock, respectively. A *MC*_*X*_ > 0 indicates that element X is enriched in the soil horizon relative to the parent rock and Ti, while a *MC*_*X*_ < 0 indicates X is leaching or migration in the soil horizon.

#### Statistical analysis methods

One-way ANOVA and a mean comparison according to the LSD (*p* < 0.05) were used to evaluate the differences in physicochemical properties among landscape slope positions and soil profile horizons. Statistical analyses were conducted using SPSS 19.0. The distribution map of the sampling sites was drafted using ArcGIS 10.8, and the other diagrams were created using Excel 2016 and Origin 9.3.

## Results

### Soil morphology

As shown in Table 2, the profiles with the pattern of A–C horizons were mainly concentrated at the summit and shoulder of the hillslope, the profile at the backslope and footslope was the A–B–C horizon, and the toeslope was the A–B horizon within the excavation depth. From the summit to the toeslope, the soil thickness increased significantly with the change in the soil profile configuration from 16.50 to 93.60 cm (*p* = 0.000). In the study area, there were three types of soil colour hues, including 10R, 2.5YR, and 5YR. The soil texture of horizon C was loam, whereas that of horizon B was mostly loam and clay loam. The soil texture of horizon A was loam to clay loam, from the summit to the toeslope (Table 3). The main soil structure of each horizon was blocky or/and granular, and the organic matter accumulation in the surface horizon, combined with mechanical ploughing, loosened the soil. The presence of a granular structure in the soil, typically characterized by loose porosity and good permeability, was generally beneficial for improving soil structure. The cohesiveness of the soil in horizon A was non-sticky, slightly sticky, and sticky from the summit to the toeslope. The cohesiveness of horizon B was mostly sticky, while that of horizon C was mostly non-sticky. Due to human cultivation or mechanical tillage, there was a small amount of intrusive material in the soil, mainly consisting of brick, tile debris, and a small amount of coal cinder. The parent rock of mudstone soil was sedimentary rock, most lacustrine facies sedimentary rock deposited during the Jurassic and Cretaceous periods, resulting in the presence of a small number of shells in the soil.

**Table 3:** The bulk density, porosity, and particle size distribution of soil profiles at different slope positions.

Slope position	Horizon	Bulk density (g cm^−3^)	Porosity (%)	Particle size distribution (%)		
				Clay	Silt	Sand
Summit	A	1.35 ± 0.09	46.36 ± 3.44	14.26 ± 2.23	47.14 ± 8.51	38.60 ± 9.37
	C	–	–	10.99 ± 3.73	40.21 ± 1.88	48.80 ± 2.95
Shoulder	A	1.37 ± 0.11	45.5 ± 4.82	17.25 ± 2.72	44.65 ± 3.33	38.09 ± 4.95
	B	1.38 ± 0.03	46.97 ± 1.47	19.59 ± 3.60	40.52 ± 3.95	39.90 ± 2.00
	C	–	–	10.39 ± 1.88	39.26 ± 1.53	50.35 ± 3.14
Backslope	A	1.36 ± 0.12	45.41 ± 5.56	21.81 ± 2.99	41.13 ± 3.31	37.07 ± 5.52
	B	1.48 ± 0.14	42.41 ± 5.20	22.14 ± 4.08	39.90 ± 5.30	37.96 ± 5.89
	C	–	–	10.54 ± 1.63	38.96 ± 0.94	50.49 ± 1.93
Footslope	A	1.43 ± 0.04	43.32 ± 1.80	30.82 ± 4.19	39.33 ± 1.96	29.85 ± 4.01
	B	1.52 ± 0.09	41.33 ± 3.72	29.13 ± 3.64	39.87 ± 3.54	31.00 ± 5.22
	C	–	–	10.13 ± 1.52	39.60 ± 0.98	50.27 ± 2.11
Toeslope	A	1.53 ± 0.16	39.64 ± 6.09	36.47 ± 3.47	38.79 ± 1.96	24.74 ± 4.20
	B	1.60 ± 0.09	37.72 ± 3.80	36.54 ± 1.28	38.91 ± 3.15	24.55 ± 3.87

### Soil physical and chemical properties

The bulk density and porosity of the soil profiles at different slope positions were showed in Table 3. The bulk density of horizons A and B of the soil profiles gradually increased from the summit to the toeslope. Additionally, the bulk density of horizon A was found to be lower than that of horizon B at all slope positions. The porosity in horizons A and B of the soil profiles also decreased gradually from the summit to the toeslope. The porosity of horizon A was higher than that of horizon B. This was primarily due to clays and oxides (such as iron and aluminum oxides) were leached downward and accumulated in the horizon B under the action of percolating water, leading to a decrease in porosity. Additionally, the topsoil was typically rich in organic matter and had higher porosity compared to the subsoil. As indicated in Table 3, the content of sand and silt fractions in horizons A and B decreased gradually from the summit to the toeslope. Conversely, the clays fraction content in horizons A and B increased gradually from the summit to the toeslope.

**Figure 3:**
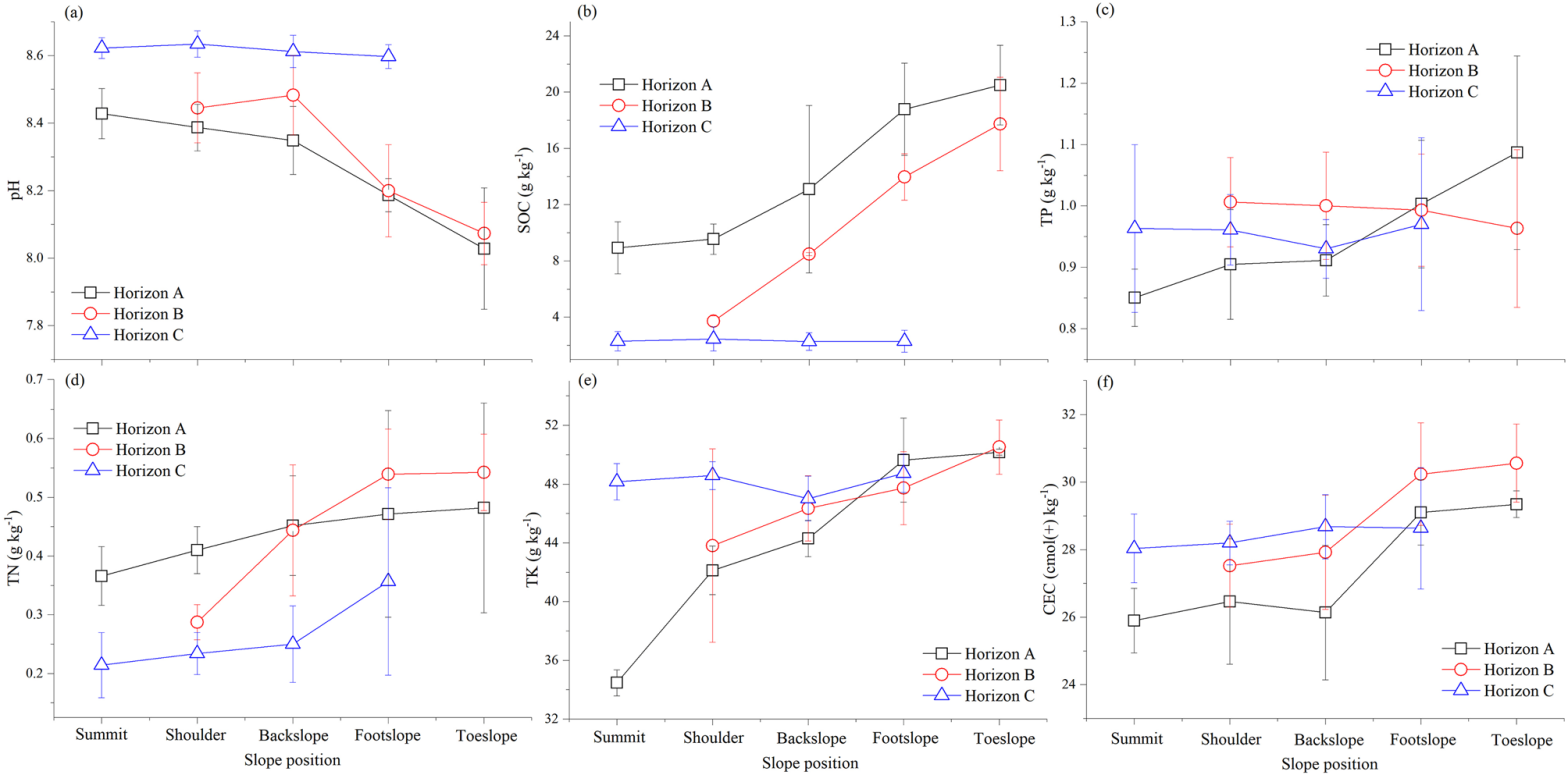
The pH (**a**), SOC (**b**), TP (**c**), TN (**d**), TK (**e**), and CEC (**f**) of soil profiles at different slope positions. SOC, soil organic carbon; TP, total phosphorus; TN, total nitrogen; TK, total potassium; CEC, cation exchange capacity.

**Figure 4:**
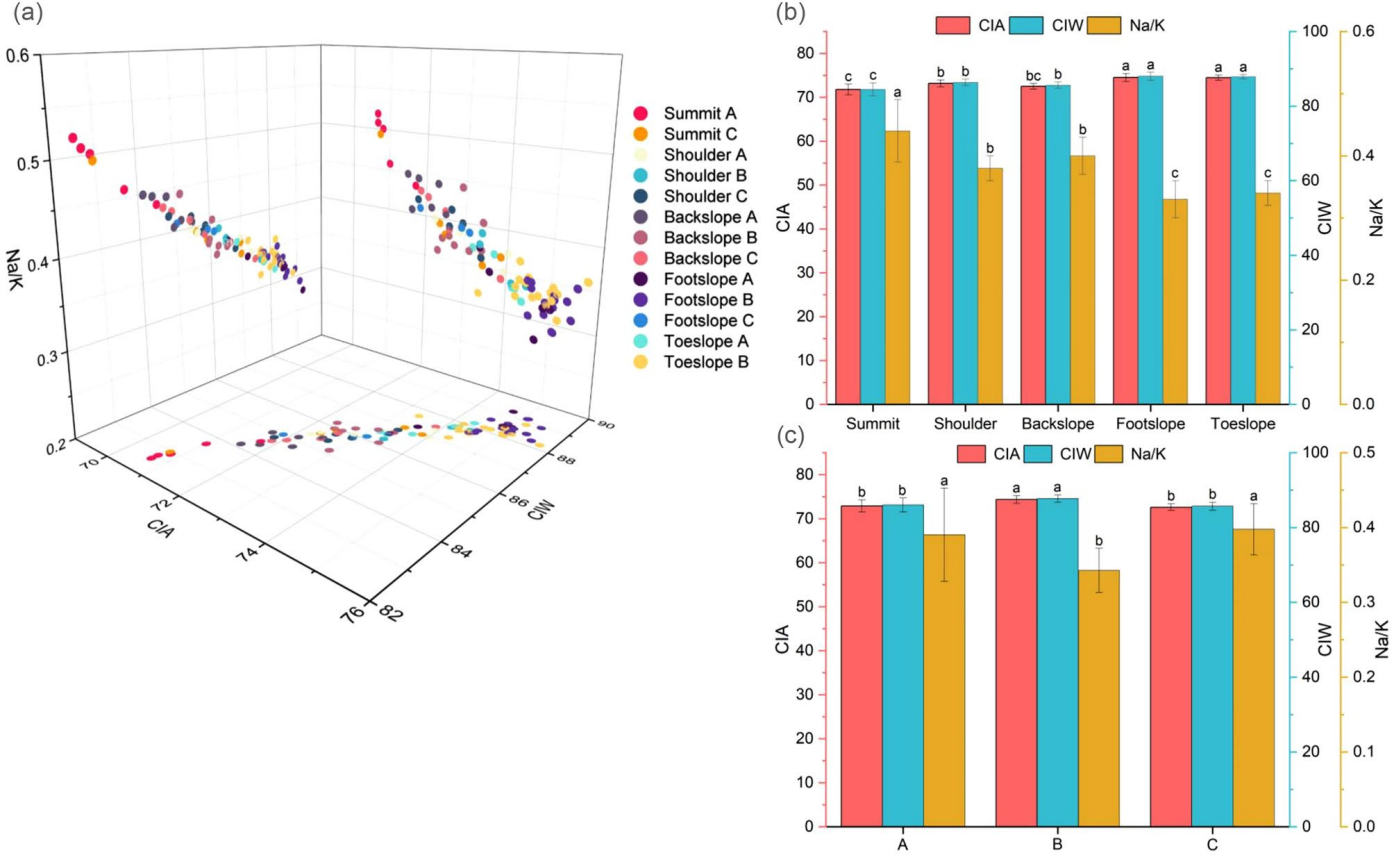
Chemical weathering parameters of soil profiles (**a**) and their means (**b**) at different landscape positions, and means at the A, B, and C horizons (**c**).

As shown in Fig. 3, the pH of the different horizons showed an order of C > B > A, and with a decreasing trend from the summit to the toeslope. The SOC content in horizon C was low, with no significant difference among the different slope positions (*p* = 0.978). The SOC content in horizons A and B gradually increased with decreasing slope elevation, and the difference in SOC above and below the backslope was significant (*p* = 0.000). The accumulation of SOC in horizon A at the footslope and toeslope was mainly due to soil erosion, cultivation and fertilisation, while the accumulation of SOC in horizon B was mainly caused by detachment, transportation, accumulation, and burial of deep soil in the higher topography. The hilly terrain was conducive to the accumulation of SOC content at the footslope and toeslope. Part of the P in the soil came from the parent rock, whereas the other part came from the application of chemical fertilisers and plant decomposition. Although phosphorus was generally considered to be relatively immobile in the soil, it could also be redistributed through the erosion of phosphorus-containing soil particles, dissolution into runoff and migration along the slope, and leaching into the ground. Therefore, the variation in phosphorus content was observed across different slope positions and profile levels during the soil formation process (Fig. 3). Total phosphorus (TP) in the soil at the summit of the slope was significantly lower than that in the toeslope (*p* = 0.043). The TP in the soil above the backslope was in the order of horizon B > C > A, whereas that in the footslope and toeslope was A > B > C. The TN content from the summit to the toeslope increased with decreasing slope elevation, and there was a significant difference above and below the backslope (*p* = 0.000), indicating that there was TN accumulation at the foot and toeslope. In general, the TN above the backslope was in the order of horizon A > B > C of the soil profile, whereas that of the footslope and toeslope was horizon B > A > C (Fig. 4). The total potassium (TK) content from the summit to the toeslope gradually increased with decreasing slope elevation. There was a significant difference between the summit, shoulder of the slope and footslope, and toeslope, indicating that K in the soil accumulated at a lower topographic position (*p* = 0.000). According to the different horizons, the TK in the soil above the backslope was in the order of horizon C > B > A, whereas that at the footslope was horizon A > C > B (Fig. 3). There were differences in the CEC content among the different soil horizons. The average CEC contents in horizons A, B, and C were 27.37, 29.67, and 28.36 cmol(+) kg^−1^, respectively. These results indicated that cations migrated and leached in horizon A, accumulating and becoming enriched in horizon B. The CEC above the backslope followed the order of horizon C > B > A, while below the backslope it was B > A > C.

As shown in Table 4, a noticeable negative correlation was found between sand content and SOC, TN, TK, CEC, and bulk density, and a significant positive correlation was observed between sand content and pH and porosity (*p* < 0.05). There was a significant negative correlation between silt content and the levels of SOC, TN, TP, TK, and CEC, as well as a significant positive correlation between silt content and pH (*p* < 0.05). Additionally, there was a negative correlation between clay content and pH and porosity, but a significant positive correlation between clay content and SOC, TN, TK, CEC, and bulk density (*p* < 0.05).

**Table 4:** Pearson’s correlation coefficients of soil physical and chemical properties.

	SP	pH	SOC	TN	TP	TK	CEC	BD	Porosity	Sand	Silt	Clay
SP	1											
pH	− 0.328**	1										
SOC	0.170	− 0.605**	1									
TN	0.172	− 0.530**	0.688**	1								
TP	0.079	− 0.221	0.156	0.191	1							
TK	0.419**	− 0.609**	0.500**	0.496**	0.373**	1						
CEC	0.469**	− 0.422**	0.532**	0.433**	0.173	0.564**	1					
BD	0.400**	− 0.354**	0.382**	0.363**	0.088	0.522**	0.436**	1				
Porosity	− 0.323**	0.357**	− 0.422**	− 0.370**	− 0.054	− 0.478**	− 0.354**	− 0.977*	1			
Sand	− 0.255*	0.531**	− 0.618**	− 0.428**	− 0.086	− 0.515**	− 0.500**	− 0.557**	0.565**	1		
Silt	− 0.265*	0.340**	− 0.277*	− 0.300*	− 0.275*	− 0.457**	− 0.356**	− 0.100	0.054	− 0.161	1	
Clay	0.383**	− 0.680**	0.725**	0.562**	0.232	0.730**	0.661**	0.573**	− 0.555**	− 0.839**	− 0.402**	1

SP, slope position; BD, bulk density; *, *p* ≤ 0.05; **, *p* ≤ 0.01.

### Geochemical composition and chemical weathering indices

In general, the footslope and toeslope of the hillslope showed accumulation of Al, Fe, Mg, and K, and leaching loss of Ca and Na compared with the summit and shoulder of the hillslope. There was a leaching loss of K in horizon A at the summit of the hillslope, while the variation in elements in each horizon at the shoulder of the hillslope was not obvious (Table 5).

**Table 5:** Geochemical element contents of soil profiles at different positions.

Position	Horizon	n	SiO_2_ (%)	Al_2_O_3_ (%)	TFe_2_O_3_ (%)	MgO (%)	CaO (%)	Na_2_O (%)	K_2_O (%)	TiO_2_ (%)	MnO (%)	P_2_O_5_ (%)
Summit	A	5	53.33 ± 1.40	13.70 ± 0.26	5.63 ± 0.16	2.29 ± 0.10	7.65 ± 0.33	0.84 ± 0.03	2.63 ± 0.09	0.62 ± 0.01	0.10 ± 0.00	0.16 ± 0.00
	C	5	51.86 ± 2.31	14.61 ± 0.98	6.07 ± 0.41	2.46 ± 0.17	7.70 ± 0.22	0.74 ± 0.07	2.84 ± 0.17	0.63 ± 0.02	0.10 ± 0.01	0.15 ± 0.01
	Summit (mean)	10	52.59 ± 1.96a	14.15 ± 0.83c	5.85 ± 0.37c	2.38 ± 0.16c	7.67 ± 0.27a	0.79 ± 0.07a	2.74 ± 0.17d	0.63 ± 0.01c	0.10 ± 0.01a	0.16 ± 0.01c
Shoulder	A	6	52.18 ± 0.42	14.48 ± 0.72	5.96 ± 0.22	2.47 ± 0.11	7.33 ± 0.17	0.70 ± 0.03	2.80 ± 0.08	0.62 ± 0.01	0.10 ± 0.01	0.19 ± 0.01
	B	4	52.01 ± 0.66	14.96 ± 0.28	6.13 ± 0.09	2.52 ± 0.11	7.16 ± 0.11	0.68 ± 0.06	2.84 ± 0.05	0.63 ± 0.01	0.10 ± 0.01	0.16 ± 0.01
	C	5	51.84 ± 1.34	14.62 ± 0.51	6.07 ± 0.27	2.49 ± 0.13	7.64 ± 0.44	0.74 ± 0.03	2.83 ± 0.11	0.63 ± 0.01	0.10 ± 0.01	0.15 ± 0.00
	Shoulder (mean)	15	52.02 ± 0.83ab	14.66 ± 0.56b	6.04 ± 0.21bc	2.49 ± 0.11b	7.39 ± 0.33ab	0.70 ± 0.04b	2.82 ± 0.08cd	0.63 ± 0.01c	0.10 ± 0.01a	0.17 ± 0.02abc
Backslope	A	6	53.21 ± 0.61	14.51 ± 0.34	6.02 ± 0.11	2.58 ± 0.06	7.04 ± 0.14	0.79 ± 0.03	2.82 ± 0.07	0.64 ± 0.01	0.10 ± 0.00	0.18 ± 0.01
	B	8	52.82 ± 0.63	14.81 ± 0.38	6.21 ± 0.18	2.59 ± 0.08	6.89 ± 0.39	0.73 ± 0.03	2.89 ± 0.14	0.63 ± 0.01	0.10 ± 0.01	0.16 ± 0.01
	C	5	51.78 ± 0.80	14.66 ± 0.79	6.09 ± 0.43	2.50 ± 0.30	7.47 ± 0.83	0.74 ± 0.03	2.85 ± 0.16	0.63 ± 0.02	0.10 ± 0.01	0.15 ± 0.01
	Backslope (mean)	19	52.67 ± 0.85a	14.68 ± 0.49b	6.12 ± 0.25c	2.56 ± 0.16ab	7.09 ± 0.53b	0.75 ± 0.04c	2.85 ± 0.12bc	0.64 ± 0.01c	0.10 ± 0.01a	0.16 ± 0.01bc
Footslope	A	6	53.07 ± 0.79	15.37 ± 0.85	6.43 ± 0.37	2.60 ± 0.08	5.07 ± 0.19	0.63 ± 0.04	2.96 ± 0.19	0.67 ± 0.02	0.10 ± 0.01	0.19 ± 0.01
	B	15	52.43 ± 0.51	15.80 ± 0.45	6.56 ± 0.26	2.64 ± 0.04	5.04 ± 0.20	0.63 ± 0.02	2.96 ± 0.15	0.67 ± 0.02	0.10 ± 0.01	0.17 ± 0.01
	C	3	51.73 ± 1.71	14.67 ± 0.39	6.07 ± 0.07	2.51 ± 0.10	7.38 ± 0.70	0.74 ± 0.02	2.85 ± 0.02	0.63 ± 0.01	0.10 ± 0.01	0.15 ± 0.00
	Footslope (mean)	24	52.50 ± 0.85a	15.55 ± 0.67a	6.46 ± 0.31a	2.62 ± 0.07a	5.34 ± 0.83c	0.64 ± 0.05d	2.95 ± 0.15ab	0.66 ± 0.02a	0.10 ± 0.01a	0.17 ± 0.02ab
Toeslope	A	5	52.20 ± 0.71	15.45 ± 0.49	6.45 ± 0.35	2.61 ± 0.05	6.06 ± 0.25	0.68 ± 0.03	2.98 ± 0.12	0.66 ± 0.01	0.10 ± 0.01	0.18 ± 0.01
	B	16	51.79 ± 0.55	15.52 ± 0.47	6.38 ± 0.34	2.59 ± 0.08	6.43 ± 0.32	0.64 ± 0.03	2.91 ± 0.09	0.65 ± 0.02	0.10 ± 0.01	0.17 ± 0.01
	Toeslope (mean)	21	51.88 ± 0.60b	15.50 ± 0.47a	6.40 ± 0.33a	2.59 ± 0.07a	6.34 ± 0.34d	0.65 ± 0.03d	2.93 ± 0.10a	0.65 ± 0.02b	0.10 ± 0.01a	0.17 ± 0.01a
Horizon A (mean)		28	52.80 ± 0.92a	14.71 ± 0.84b	6.10 ± 0.39b	2.52 ± 0.14b	6.61 ± 0.99b	0.72 ± 0.08a	2.84 ± 0.17b	0.64 ± 0.02ab	0.10 ± 0.01a	0.18 ± 0.01a
Horizon B (mean)		43	52.23 ± 0.67b	15.43 ± 0.57a	6.39 ± 0.30a	2.60 ± 0.08a	6.10 ± 0.86c	0.66 ± 0.05b	2.92 ± 0.12a	0.65 ± 0.02a	0.10 ± 0.01a	0.16 ± 0.01b
Horizon C (mean)		18	51.81 ± 1.47b	14.64 ± 0.67b	6.08 ± 0.32b	2.49 ± 0.18b	7.57 ± 0.54a	0.74 ± 0.04a	2.84 ± 0.12ab	0.63 ± 0.02b	0.10 ± 0.01a	0.15 ± 0.01c

Values for different geochemical element contents in a column followed by the same lowercase letter are not significantly different at *p* < 0.05. Data are means ± SD.

As demonstrated in Table 5, Al, Fe, and Mg accumulated in horizon B, whereas the percentages of Ca and Na in horizon B were lower compared to horizons A and C. Additionally, Si exhibited a trend of A > B > C across various soil profile horizons. Generally, weathering tended to be most intense nearest the soil surface. Part of the weathering products formed in horizon A were leached downward under the action of percolating water, coupled with the weathering materials of horizon B itself, which led to the accumulation of Al, Fe and Mg in horizon B. Simultaneously, minerals with low weathering stability were gradually destroyed and reduced, while quartz with high weathering stability was enriched. This resulted in a sequential decrease in the percentage of Si from topsoil to subsoil. The leaching of Na and Ca was significant in horizons A and B (*p* < 0.05), whereas the leaching of Ca and Na was obvious in horizon B relative to horizon A in the hillslope. During the development of silicate soils, Na and Ca were leached first, followed by K, and Al and Fe were relatively enriched. The soil in this study was in the stage of leaching of Ca and Na, the leaching of soil geochemical elements was not strong, and the degree of soil development was weak. The effect of microtopography on soil chemical weathering was greater than that on the profile.

The chemical index of alteration (CIA) exhibited an increasing trend, whereas Na/K demonstrated a converse trend from summit to toeslope (Fig. 4a). The CIA values of summit, shoulder, backslope, footslope, and toeslope were 71.78, 73.17, 72.51, 74.55, and 74.55, respectively (Fig. 4b). The CIA values of horizons A, B, and C were 72.92, 74.37, and 72.61, respectively (Fig. 4c). There were significant differences in CIA values between horizons A and B, B and C (*p* = 0.000). The variation trend of chemical index of weathering (CIW) was basically the same as that of CIA. Furthermore, both CIA and CIW have similar indicators for reflecting the degree of soil weathering. The variance *F* value of the soil chemical weathering index under different factors demonstrated that the CIA, CIW, and Na/K of soil varied significantly between different horizons at various slope positions. Additionally, there was a significant interaction between slope position and horizon (Table 6). Simultaneously, the CIA, CIW, and Na/K values indicated that the soil in the study area had moderate chemical weathering, and the chemical weathering of the soil parent horizon remained at the same level from the summit to the toeslope of the hillslope. The CIA, CIW, and Na/K values of horizons A and B showed a trend of initially increasing and then decreasing with decreasing slope elevation. Horizon B at the footslope exhibited the highest degree of development, which indicated that the eroded and weathered material stripped and transported from higher landscape positions was deposited into horizon B at the footslope duo to topographical influences.

**Table 6:** Analysis of variance *F* value of chemical weathering indicators of soils at different slope positions and horizons.

Factor	CIA		CIW		Na/K	
	*F*	Sig	*F*	Sig	*F*	Sig
Slope position	27.298	0.000	29.754	0.000	23.191	0.000
Soil genetic horizon	19.951	0.000	16.077	0.000	8.985	0.000
S × H	7.222	0.000	9.223	0.000	8.506	0.000

S × H, interaction of slope position and horizon.

**Figure 5:**
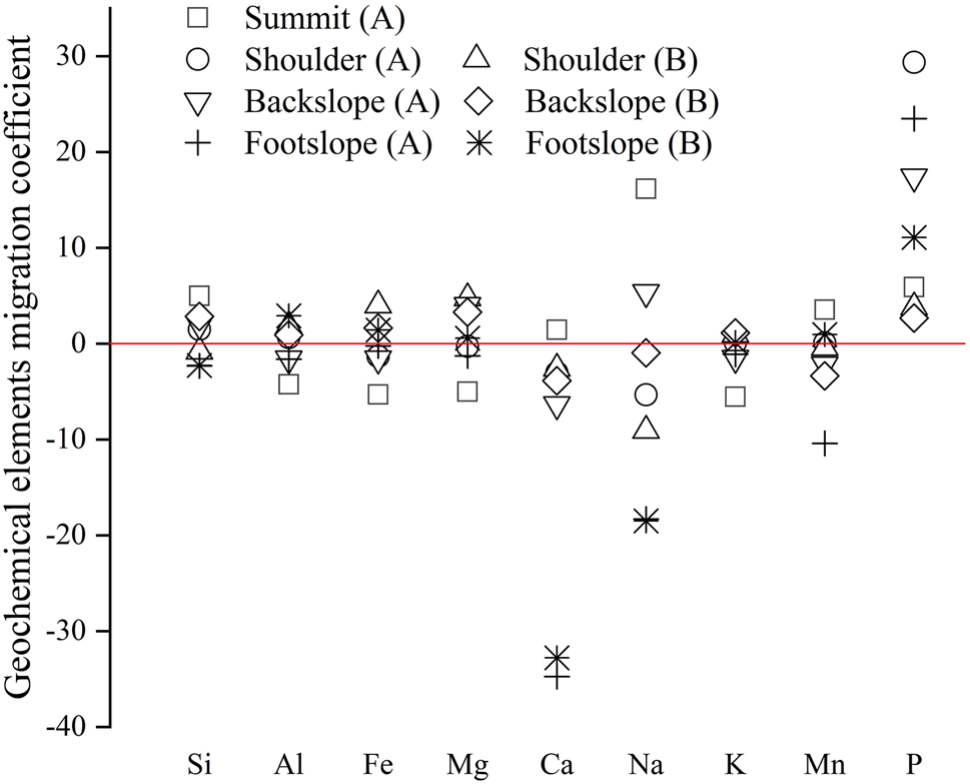
Geochemical elements migration coefficient of soil profiles at different slope positions.

As shown in Fig. 5, the mobility of the elements varied at different landscape positions. The migration directions of Ca and Na at the summit and backslope were completely opposite to that at the footslope. The average migration coefficients of Ca at the summit, backslope, and footslope were 1.43%, − 4.93%, and − 33.43%, respectively. The average migration coefficients of Na were 16.15%, 1.73%, and − 18.43%, respectively, and the migration direction changed from enrichment to leaching. Al, Fe, and Mg were first leached and then enriched from the summit to the footslope, which may be due to the higher sand content in the soil and relatively large intergranular pores, resulting in the loss of elements under the action of rainfall and underground runoff. However, in areas with relatively low terrain, like the footslope, the abundant water conditions provided the ideal environment for soil chemical weathering. This led to the leaching of Ca and Na, while Al and Fe became enriched.

### Mineralogy characteristics

**Figure 6:**
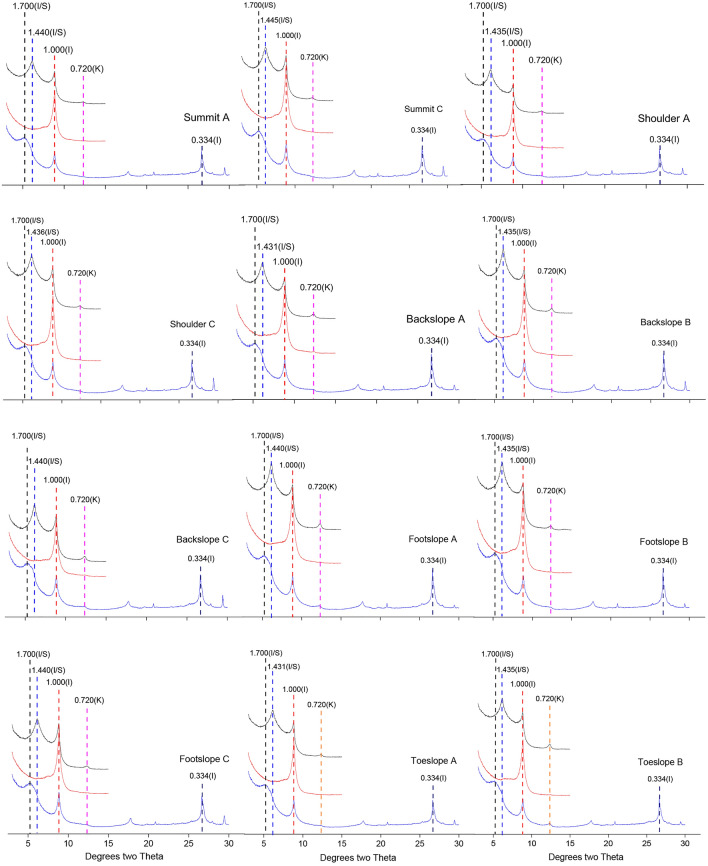
X-ray diffraction data for soils at different slope positions (A–horizon A, B–horizon B, and C–horizon C). The sequence of treatments is represented by three different colours: the black (top), red (middle position), and blue (bottom) curves represent the air-dried mount (AD) samples, samples heated at 550 °C (K550), and glycol solvated (EG) samples, respectively. I: illite; K: kaolinite; I/S: illite/smectite mixed-horizon.

The mineral composition of the soils was analysed using X-ray diffraction (Fig. 6). There was an evident mineralogical similarity between the soils at different slope positions and horizons. The mudstone soils in this study were primarily composed of illite, kaolinite, and an illite/smectite mixed horizon. The mineral composition of the soils closely resembled that of their parent rock. These results indicated that the clay minerals in the soils originated mostly from the parent material and that they were only slightly influenced by soil-forming processes.

## Discussion

### Effect of microtopography on the physical and chemical weathering of the soil at different slope positions

The spatial heterogeneity in particle size distribution, pH, SOC, elements, and CEC across landscape positions were influenced by microtopography^43,44^. A study conducted on the Moody and Nora soil system toposequences formed in calcareous loess in eastern Nebraske showed that sand, silt, and pH exhibited an overall increasing trend from the top to the bottom of the slope. Inversely, clay particles, organic matter (excluding toeslope), and CEC showed a decreasing trend^43^. This is contrary to our findings. The differences in particle size distribution among landscape positions may be attributed to soil erosion and the downward migration of clay particles under the action of percolating water^10^. In the present study, the texture of horizon A primarily ranged from loam to clay loam. On one hand, due to the influence of surface runoff, partial clay particles migrated along the slope and accumulate at the bottom^45–47^. On the other hand, under the action of percolating water, the smallest clay particles were leaching downward into the underlying horizons, resulting in an increase in the percentage of clay in horizon B (Table 3). Zhong et al.^48^ also showed that the sand content and mineral sand of 0.25–2 mm in the soil, developed by shale and mudstone, decreased from the top to the foot of the slope. In contrast, the clay content and aggregate content of 0.25–2 mm increased. The opposite trend in pH observed may be attributed to the topsoil analyzed by Brubaker et al.^43^ being rich in Ca, and the Mg content increased continuously from the summit to toeslope. Although some leaching occurs during the downslope migration, a considerable portion still migrates to the bottom of the slope, resulting in enrichment. The decrease in organic matter from summit to footslope may also contribute to the increase in pH. In this study, the continuous enrichment of Al, leaching of Ca and accumulation of organic matter from the summit to toeslope resulted in the decrease of soil pH. This was further illustrated by the significant negative correlation between pH and aluminum oxide and SOC in Table 4. The increase in SOC from the summit to the toeslope may be related to soil erosion. A study of toposequences in olive grove in the Mediterranean also indicated that erosion caused the transfer of organic matter from the summit to the toeslope^44^. Ouyang et al.^17^ conducted a study on the CEC value of soils developed in granite, slate, limestone, sandstone and quaternary red clay in mid-subtropical Hunan Province, China. This study demonstrated that the CEC value was higher on the footslope compared to the backslope. The findings of the present study corroborate these results, showing that the CEC values on the footslope and toeslope were higher compared to other landscape positions. This higher CEC value was attributed to the accumulation of SOC and clay particles, as indicated by a significant positive correlation in Table 4.

The degree of soil chemical weathering varied with changes in the slope position and soil depth. Topographic changes caused differences in chemical weathering of the soil profile, mainly because the soil at the top and shoulder of the slope was mostly developed under natural conditions and rarely affected by human beings. In these areas, soil weathering mainly depended on the changes in natural climate conditions and the effect of soil organisms. Conversely, at the footslope and other relatively low topographic locations, soil moisture is retained, providing ideal moisture conditions for chemical weathering to occur. Meanwhile, the soil on the footslope and toeslope was affected by cultivation practices. Irrigation alters the soil moisture status, tillage changes soil aeration conditions^49^, and fertilisation increases soil nutrient elements. These factors contribute to soil mineral weathering and nutrient leaching, while also intensifying soil erosion^50–52^. Therefore, soil erosion caused by tillage promotes chemical weathering in the soil.

### The redistribution of flow and materials by microtopography resulted in the difference of pedogenic characteristics at different slope positions

Water runs through the entire process of soil occurrence and development. It plays a crucial role in soil formation by serving as the primary medium for transporting solids and ions within the soil^53^. Microtopography dominates surface hydrological processes and affects water redistribution^54,55^, leading to variations in the physicochemical properties of soil at different slope positions^15^. In low-lying areas, the regolith is typically more extensively weathered, and the soil profile is developed with greater intensity due to the accumulation of flowing water. This water saturation also restricts drainage and ventilation, limiting the weathering of certain minerals and the decomposition of organic matter^10^. Therefore, even within a microdomain of tens of meters, the soil of the same genus also forms different textures owing to the various properties. In this study, the physicochemical properties of the soil varied with the change in the slope position, particularly between the soil above and below the backslope, with differences also seen among different horizons. The mudstone parent rock, exposed at the summit of the hilly mountainous region caused by erosion, is rich in clay minerals and has a strong water absorption capacity. Therefore, it is easily weathered physically under the influence of moist heat expansion. Consequently, stony subsoil is frequently formed at or near the summit of the slope^48^. Obi et al.^46^ showed that sand content is affected by rainfall and infiltration. Excessive rainfall, when surpassing infiltration, leads to the redistribution of sand within the slope and soil horizon. This particular occurrence is commonly witnessed during thunderstorm weather conditions, subsequently compromising the stability of the soil surface on the slope^56,57^. Additionally, the study findings indicate that the level of chemical weathering in horizon A at the footslope and toeslope was comparatively lower than that of horizon B. This disparity can be attributed to the redistribution of soil materials brought about by microtopography^3^. This finding contradicts the results of other toposequences, which indicated that horizon A (or topsoil) undergoes greater chemical weathering than horizon B (or subsoil)^21,58^. This could be due to severe soil and water loss in hilly mountainous regions, where materials transported by upper erosion are deposited at the footslope and toeslope. Long-term contact between water and sediment leads to further chemical action, resulting in soil with a high organic matter content and fine texture. This soil is then buried by a new round of denudation accumulation and self-weathered soil, eventually becoming a B-horizon with a higher degree of development than the topsoil.

## Conclusion

In this study, we investigated the influence of microtopography on the morphological characteristics, physiochemistry, and geochemical attributes of the profiles. The results indicated that the morphological characteristics of the mudstone soil profile were mainly inherited and affected by the parent material. From the summit to the toeslope, the profile configuration of the mudstone soil changed from A–C to A–B–C, and the thickness of the soil increased significantly. The bulk density, clay fraction, soil organic matter, TN, TP, TK, and CEC increased from the summit to the toeslope of the hillslope, whereas the pH, porosity, sand, and silt fraction decreased. The total contents of Ca and Na at the summit, backslope, and footslope decreased due to the continuous leaching along the downslope direction, while the total contents of Al, Fe and Mg showed an opposite trend. The degree of soil development was relatively weak at the summit and shoulder of the hillslope, but higher at the footslope and toeslope. Microtopography may influence physicochemical properties, chemical weathering, and redistribution of water and materials, leading to variations in pedogenic characteristics at different slope positions.

## Data Availability

All raw data can be obtained from the corresponding authors on request.
